# Quantitative not qualitative histology differentiates aneurysmal from nondilated ascending aortas and reveals a net gain of medial components

**DOI:** 10.1038/s41598-021-92659-1

**Published:** 2021-06-23

**Authors:** Sameh Yousef, Nana Matsumoto, Issam Dabe, Makoto Mori, Alden B. Landry, Shin-Rong Lee, Yuki Kawamura, Chen Yang, Guangxin Li, Roland Assi, Prashanth Vallabhajosyula, Arnar Geirsson, Gilbert Moeckel, Jay D. Humphrey, George Tellides

**Affiliations:** 1 Section of Cardiac Surgery, Department of Surgery, Yale School of Medicine, 10 Amistad Street 337B, New Haven, CT 06520 USA; 2 Department of Pathology, Yale School of Medicine, New Haven, CT USA; 3 Department of Breast and Thyroid Surgery, Peking University Shenzhen Hospital, Shenzhen, Guangdong Province China; 4 Vascular Biology and Therapeutics Program, Yale School of Medicine, New Haven, CT USA; 5 Veterans Affairs Connecticut Healthcare System, West Haven, CT USA; 6 Department of Biomedical Engineering, Yale School of Engineering and Applied Science, New Haven, CT USA

**Keywords:** Cell biology, Structural biology, Cardiology, Diseases, Medical research, Pathogenesis

## Abstract

Medial degeneration is a common histopathological finding in aortopathy and is considered a mechanism for dilatation. We investigated if medial degeneration is specific for sporadic thoracic aortic aneurysms versus nondilated aortas. Specimens were graded by pathologists, blinded to the clinical diagnosis, according to consensus histopathological criteria. The extent of medial degeneration by qualitative (semi-quantitative) assessment was not specific for aneurysmal compared to nondilated aortas. In contrast, blinded quantitative assessment of elastin amount and medial cell number distinguished aortic aneurysms and referent specimens, albeit with marked overlap in results. Specifically, the medial fraction of elastin decreased from dilution rather than loss of protein as cross-sectional amount was maintained while the cross-sectional number, though not density, of smooth muscle cells increased in proportion to expansion of the media. Furthermore, elastic lamellae did not thin and interlamellar distance did not diminish as expected for lumen dilatation, implying a net gain of lamellar elastin and intralamellar cells or extracellular matrix during aneurysmal wall remodeling. These findings support the concepts that: (1) medial degeneration need not induce aortic aneurysms, (2) adaptive responses to altered mechanical stresses increase medial tissue, and (3) greater turnover, not loss, of mural cells and extracellular matrix associates with aortic dilatation.

## Introduction

The aortic wall consists of three layers, a predominant media between a relatively thin intima and adventitia, each with specific cell types, structure and function. The media consists of concentric lamellae with a repeating pattern of elastic fibers alternating with collagen bundles, smooth muscle cells (SMC), and glycosaminoglycans^1^. The microstructural architecture of the media appears pristine in aortas of healthy young individuals, but typically deteriorates with age^2^. Medial degeneration is also prominent in aortic aneurysm disease and is characterized by accumulation of mucoid extracellular matrix (ECM), fragmentation and loss of elastic fibers, disorganization, dysfunction and loss of SMC, and collagen remodeling, often fibrotic^3^. A degenerated media is commonly thought to play a central role in aortic aneurysm formation by allowing a weakened vessel wall to dilate passively, a simplistic model of material failure unable to maintain normal aorta size against hemodynamic loads^4^. Yet aneurysmal aortas distend less with physiological pressure loading, both clinically and in numerous experimental models of genetically compromised SMC and ECM as well as after acute digestion of elastin in normal aortas^5,6^. A refinement of the hypothesis is that the extant ECM, especially stiff collagen fibers, are actively remodeled (via continual degradation, neosynthesis, reorientation, and cross-linking of subunits under conditions of altered wall stresses) by the intramural cells^7^. That is, the aortic wall has inherent material stiffness with limited capacity to dilate unless the underlying microstructure is fundamentally re-organized by viable and responsive vascular cells.

The diagnosis of medial degeneration is made microscopically. Alterations in vessel wall structure are clearly visible using standard histology. This allowed early pathologists to ascribe ground substance accumulation, loss of elastic fibers, and death of SMC as causes of aortic aneurysms^8–10^. Yet, observations at a microscopic level may not inform of global effects on the vessel wall if there are changes in medial mass. For example, local accumulation of collagen and glycosaminoglycans may give the appearance of loss of elastin or SMC under magnification, but this dilutional effect may be misleading if the overall presence of these components has not decreased. The phenomenon of elastin dilution was first demonstrated by biochemical assessment of circumferential protein expression in 1-cm rings of abdominal aortic aneurysms with 8-fold greater mass than similar size rings of nondilated aortas^11^. A bias toward interpreting loss of material in histological sections can be compounded by focusing on unrepresentative fields that are the most abnormal, recognizing that localized defects can set into motion a global abnormality. To objectively determine the extent of medial degeneration in thoracic aortic aneurysms versus nondilated referent specimens, we contrasted qualitative (semi-quantitative) histopathological examination with quantitative histomorphometric assessment that included derived parameters based on the cross-sectional area of the media. To minimize sampling variation and observer bias, the specimens were collected prospectively using standardized tissue location and orientation and the investigators evaluating the histological slides were blinded to the clinical diagnosis. We find that histopathological assessment of medial degeneration does not differentiate aneurysmal from nondilated aortas, while quantitative measures show a net gain of medial components in aneurysms despite structural disorganization.

## Results

### Subject characteristics

Tissue samples were collected from the right anterolateral, mid-ascending aorta of patients with thoracic aortic aneurysms undergoing surgical repair (*n* = 18) or from organ donors with nondilated aortas (*n* = 17). The diameter of aortic aneurysms ranged from 4.5 to 6.3 cm and referent specimens from 2.5 to 3.7 cm. After indexing to age, sex, and body surface area, aneurysmal aortas all exceeded 2 standard deviations (SD) of expected diameter, whereas nondilated aortas were all within 2 SD of expected diameter. Subjects with aneurysmal aortas were older, more likely to be Caucasian, and with a greater incidence of bicuspid aortic valve, aortic valve insufficiency, hyperlipidemia, and use of lipid lowering agents (Table 1). Differences in other determinants that may influence aorta size, such as sex, body surface area, hypertension, smoking, diabetes, aortic valve stenosis, aortic atherosclerosis, and use of antihypertensive agents, did not reach statistical significance.

**Table 1:** Subject characteristics.

	Nondilated	Aneurysm	*P* value
	*n* = 17	*n* = 18	
Age (years)	48.6 ± 18.4	62.7 ± 11.0	0.0273
Sex (male)	11 (65%)	16 (89%)	0.1212
**Race**			
African American	4 (24%)	0 (0%)	0.0299
Caucasian	9 (53%)	16 (89%)	
Hispanic	4 (24%)	2 (11%)	
Body surface area (m^2^)	1.98 ± 0.24	2.06 ± 0.29	0.2584
Aorta diameter (cm)	3.18 ± 0.40	5.19 ± 0.46	< 0.0001
Aorta z-score	− 0.25 ± 1.04	5.56 ± 1.77	< 0.0001
Bicuspid aortic valve	1 (6%)	9 (50%)	0.0072
Aortic valve insufficiency	0 (0%)	11 (61%)	0.0001
Aortic valve stenosis	1 (6%)	4 (22%)	0.3377
Aortic atherosclerosis	5 (29%)	1 (6%)	0.0877
Hypertension	6 (35%)	12 (67%)	0.0943
Hyperlipidemia	2 (12%)	12 (67%)	0.0016
Diabetes mellitus	2 (12%)	3 (17%)	> 0.99
Smoking	12 (71%)	7 (39%)	0.0922
Lipid lowering agents	3 (18%)	14 (78%)	0.0006
Beta blockers	5 (29%)	9 (50%)	0.3053
Calcium channel blockers	1 (6%)	3 (17%)	0.6026
ACEI/ARB	3 (18%)	7 (39%)	0.2642

Ascending aorta specimens were procured from organ donors (nondilated) and patients undergoing aortic aneurysm repair (aneurysm). Continuous variables are represented as mean ± SD and categorical variables are represented as number of subjects and percentage of total subjects. Comparisons of continuous variables are by Mann–Whitney test and of categorical variables by Fisher’s exact test.

*ACEI* angiotensin-converting enzyme inhibitors, *ARB* angiotensin II receptor blockers.

### Qualitative histopathology does not differentiate aneurysmal from nondilated aortas

**Figure 1:**
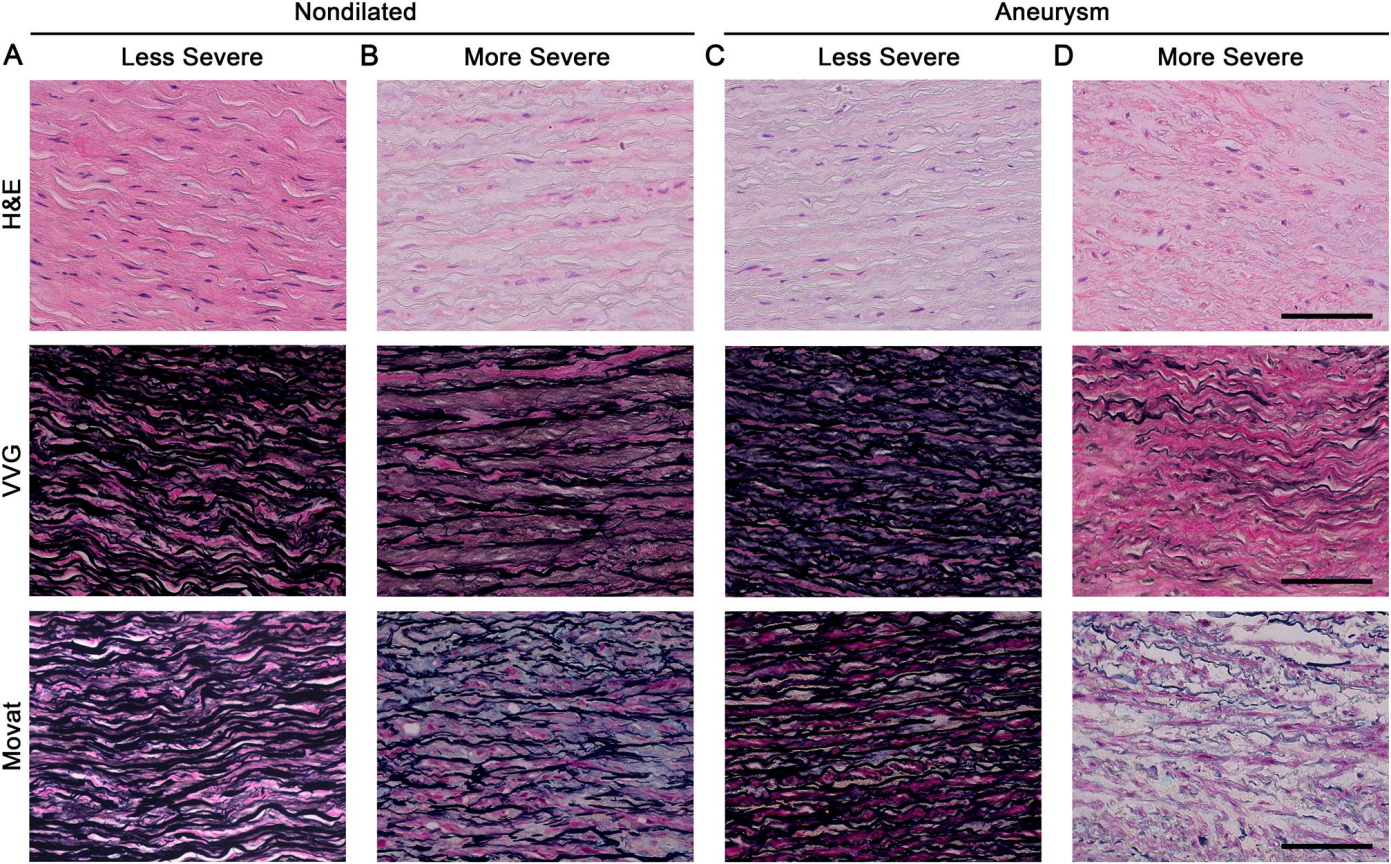
Spectrum of histological abnormalities. Transverse sections of ascending aorta specimens from organ donors (nondilated) and patients undergoing aneurysm repair (aneurysm) were stained with H&E, VVG, or Movat and graded. A wide range of abnormalities from less to more severe were found and examples are shown from (**A**) 18-year-old male with 2.6 cm aorta, (**B**) 60-year-old female with 3.2 cm aorta, (**C**) 51-year-old male with 6.5 cm aorta, and (**D**) 74-year-old male with 5.9 cm aorta. Scale bars represent 100 μm.

The tissue specimens were coded without identifying information and transverse sections were prepared with standard histological stains. Aortas of both groups showed a wide range of histological abnormalities, with severe medial degeneration in some nondilated aortas and mild medial degeneration in some aneurysmal aortas (Fig. 1). Grading of medial degeneration by an experienced pathologist blinded to the clinical diagnosis did not distinguish aneurysmal from nondilated ascending aortas (Table 2). There were no significant differences in the overall medial degeneration score or numerous individual medial degeneration criteria, such as the extent and distribution of mucoid ECM accumulation, elastic fiber fragmentation and/or loss, elastic fiber thinning, elastic fiber disorganization, SMC nuclei loss, laminar medial collapse, SMC disorganization, and medial fibrosis. Independent analyses by two pathology trainees, also blinded to the clinical diagnoses, did not find differences in overall grade or more than a single individual criterion of medial degeneration of laminar medial collapse (Supplementary Table S1). Inter-rater agreement for overall medial degeneration score among the three observers was high (Spearman’s ρ = 0.7347–1.00, *P* < 0.001). Additional blinded analyses by two investigators without pathology training also failed to differentiate aneurysmal from non-dilated aortas (data not shown). Thus, extensive overlap in abnormalities between groups prevents reliable histological diagnosis of aortic aneurysm disease.

**Table 2:** Qualitative histopathological scoring of medial degeneration.

	Nondilated	Aneurysm	*P* value
	*n* = 17	*n* = 18	
**Medial degeneration**			
Mild	6 (35%)	2 (11%)	0.2185
Moderate	4 (24%)	8 (44%)	
Severe	7 (41%)	8 (44%)	
**Mucoid ECM accumulation**			
Intralamellar			
Grade			
None	1 (6%)	0 (0%)	> 0.99
Mild	9 (53%)	10 (56%)	
Moderate	4 (24%)	5 (28%)	
Severe	3 (18%)	3 (17%)	
Distribution			
Absent	1 (6%)	0 (0%)	0.2895
Focal	5 (29%)	2 (11%)	
Multifocal	5 (29%)	5 (28%)	
Extensive	6 (35%)	11 (61%)	
Translamellar			
Grade			
None	14 (82%)	9 (50%)	0.1919
Mild	1 (6%)	4 (22%)	
Moderate	1 (6%)	4 (22%)	
Severe	1 (6%)	1 (6%)	
Distribution			
Absent	14 (82%)	9 (50%)	0.0793
Focal	0 (0%)	3 (17%)	
Multifocal	3 (18%)	6 (33%)	
**Elastic fiber fragmentation and/or loss**			
Grade			
Mild	14 (82%)	14 (78%)	> 0.99
Moderate	3 (18%)	4 (22%)	
Distribution			
Focal	9 (53%)	9 (50%)	> 0.99
Multifocal	8 (47%)	9 (50%)	
**Elastic fiber thinning**			
Grade			
None	0 (0%)	2 (11%)	0.4857
Mild	17 (100%)	16 (89%)	
Distribution			
Absent	0 (0%)	(11%)	0.4857
Focal	17 (100%)	16 (89%)	
**Elastic fiber disorganization**			
Distribution			
Focal	11 (65%)	10 (56%)	0.7332
Multifocal	6 (35%)	8 (44%)	
**SMC nuclei loss**			
Type			
Patchy	17 (100%)	18 (100%)	–
Distribution			
Frequent	17 (100%)	18 (100%)	–
**Laminar medial collapse**			
Appearance			
None	3 (18%)	7 (39%)	0.2642
Thin	14 (82%)	11 (61%)	
Distribution			
Absent	3 (18%)	7 (39%)	0.4213
Focal	11 (82%)	8 (44%)	
Multifocal	3 (18%)	3 (17%)	
**SMC disorganization**			
Distribution			
Absent	17 (100%)	18 (100%)	–
**Medial fibrosis**			
Grade			
None	8 (47%)	9 (50%)	> 0.99
Mild	9 (53%)	9 (50%)	
Distribution			
Absent	8 (47%)	9 (50%)	> 0.99
Focal	9 (53%)	9 (50%)	

Qualitative (semiquantitative) scoring of medial degeneration by an experienced pathologist of ascending aorta specimens from organ donors (nondilated) and patients undergoing aneurysm repair (aneurysm). The specimens were assessed blinded to the clinical diagnosis. Diagnostic criteria include overall medial degeneration score and several individual components of medial degeneration categorized by grade and distribution (subcategories without scores are not shown). Categorical variables are represented as score totals and percentage of total subjects and comparisons of the criteria with multiple subcategory scores are by Fisher’s exact test.

### Quantitative histomorphometry reveals decreased medial fraction but not cross-sectional presence of elastin and increased total number but not density of SMC

**Figure 2:**
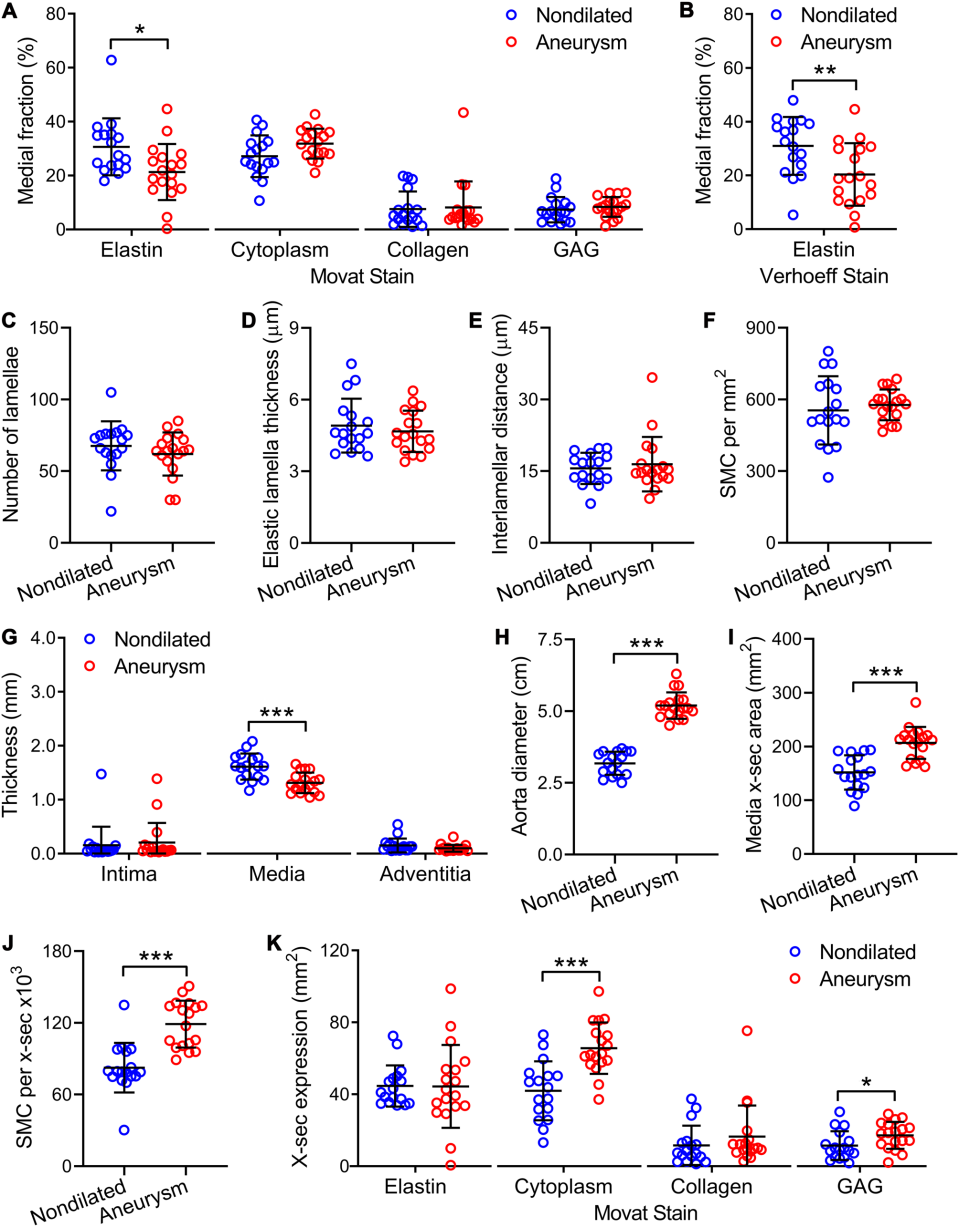
Decreased medial fraction of elastin and increased number of SMC per cross-section. Nondilated ascending aortas from organ donors and aneurysmal ascending aortas from patients undergoing surgical repair were analyzed by histomorphometry. (**A**) Image analysis of Movat stains for color components highlighting elastic fibers, cytoplasm, collagen fibers, and glycosaminoglycans (GAG) expressed as fraction of media. (**B**) Similar analysis of Verhoeff stain for additional assessment of elastin expression. Verhoeff and H&E stains were also analyzed for (**C**) number of elastic lamellae, (**D**) elastic lamella thickness, (**E**) interlamellar distance, and (**F**) density of SMC. (**G**) Thickness of intima, media, and adventitia from VVG stains and (**H**) external diameter of aortas. These primary data were used to derive (**I**) media cross-sectional (x-sec) area, (**J**) number of SMC per cross-section, and (**K**) elastin, cytoplasm, collagen, and glycosaminoglycan expression per cross-section from Movat stains. Individual data shown with bars representing mean ± SD, *n* = 17 nondilated and *n* = 18 aneurysms, *P < 0.05, **P < 0.01, ***P < 0.001 aneurysm versus nondilated, Mann–Whitney test.

Histological sections were further analyzed by investigators blinded to the clinical diagnosis until derived measurements were later calculated based on aorta size. High-power fields in the inner, mid, and outer media (*n* = 9) were arbitrarily chosen (without comparisons to other fields to select for greater or lesser pathology) and quantified for separate color signals of Movat-stained sections specific for cellular and ECM components by image analysis software. The medial fraction of elastin was decreased in aneurysms, whereas that of cytoplasm, collagen, and glycosaminoglycans were similar (Fig. 2A). Decreased medial fraction of elastin was confirmed in separate sections stained with monochromatic Verhoeff’s reagents using an alternate analysis program (Fig. 2B). Additional histomorphometry of Verhoeff-Van Gieson (VVG), Verhoeff reagent alone, and hematoxylin and eosin (H&E) stains revealed no differences in the number of elastic lamellae, elastic lamella thickness, interlamellar distance, and density of SMC (Fig. 2C–F). Despite thinning of the media, the cross-sectional area of the media in aortic aneurysms was greater because of the larger aorta diameter (Fig. 2G–I). This implied that the number of SMC per cross-section and the cross-sectional presence of cytoplasm and glycosaminoglycans were increased, but that of elastin and collagen were overall unchanged (Fig. 2J,K). These results underscore the need to assess both relative and total quantity to differentiate dilution and concentration from loss or gain of components in the context of changes in cross-sectional area.

### Interrelated quantitative measures indicate adaptive aortic wall remodeling

**Figure 3:**
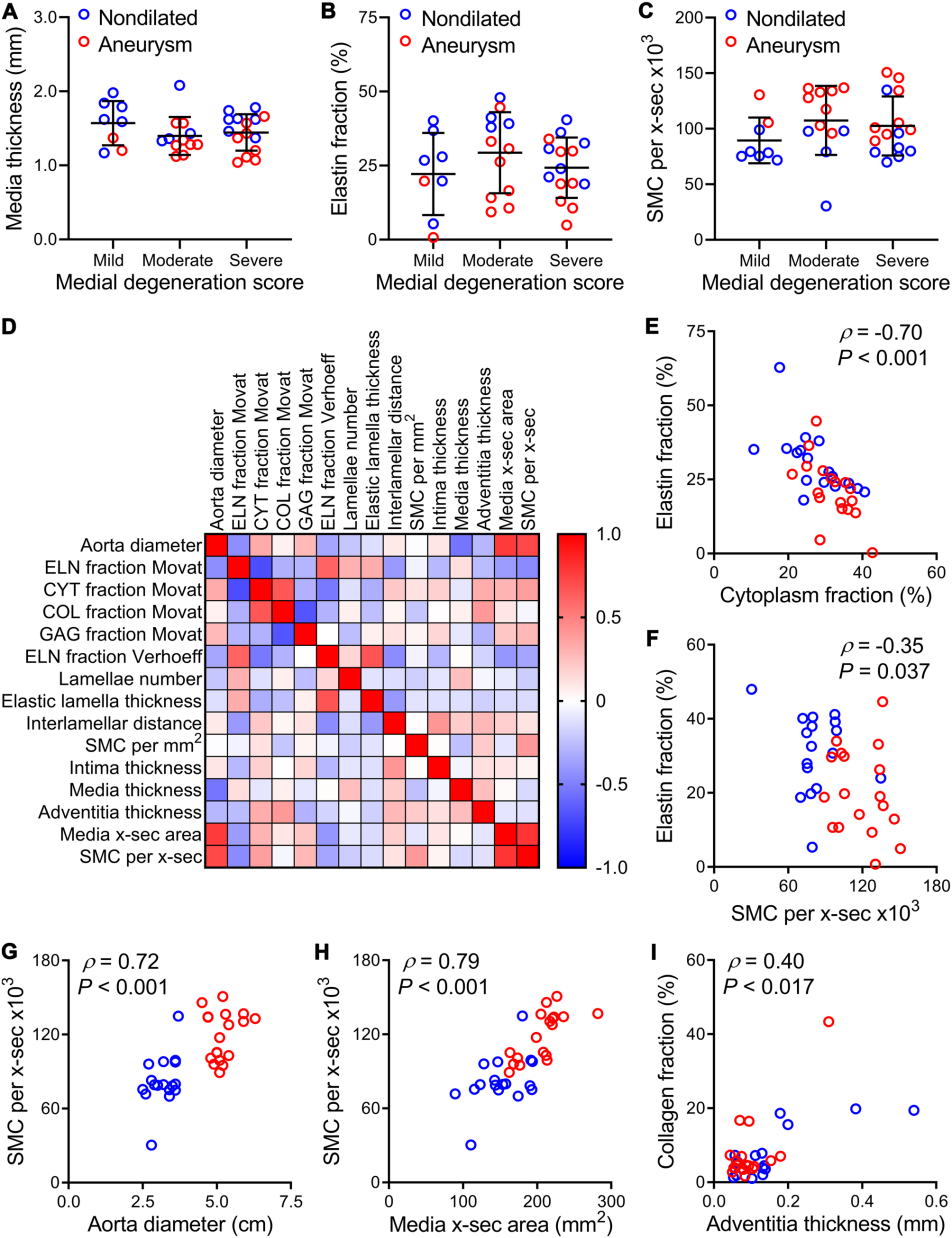
Correlation of histology data. Nondilated ascending aortas from organ donors and aneurysmal ascending aortas from patients undergoing surgical repair were analyzed by histopathology and histomorphometry. Association of medial degeneration score and (**A**) media thickness, (**B**) medial fraction of elastin by Verhoeff stain, and (**C**) number of SMC per cross-section (x-sec). (**D**) Matrix of correlation coefficients among quantitative data. Correlation of (**E**) medial fraction of elastin to cytoplasm by Movat stain, (**F**) medial fraction of elastin by Verhoeff stain to number of SMC per cross-sectional area, (**G**) number of SMC per cross-sectional area to aorta diameter, (**H**) number of SMC per cross-sectional area to media cross-sectional area, and (**I**) medial fraction of collagen by Movat stain to adventitia thickness. Individual data shown with bars representing mean ± SD, *n* = 17 nondilated and *n* = 18 aneurysms, and comparisons were by Kruskal–Wallis test with Dunn’s multiple comparisons test (**A**–**C**) or by Spearman correlation (**D**–**I**).

We examined for associations between histopathology and histomorphometric readouts. The medial degeneration score was not significantly related to several enumerated outcomes of aneurysm disease, including media thickness, medial fraction of elastin, and number of SMC (Fig. 3A–C). This finding highlighted the limited predictive ability of qualitative analysis to differentiate aneurysmal from nondilated aortas. To gain insight into mechanisms of aortic wall remodeling, we assessed for correlations among the quantified parameters (Fig. 3D and Supplementary Table S2). Specifically, the medial fraction of elastin was inversely related to that of cytoplasm and number of SMC suggesting that elastin dilution or loss drives SMC hyperplasia and hypertrophy and/or that SMC degrade elastin (Fig. 3E,F). The strongest correlates for the number of SMC was aorta diameter and media cross-sectional area, suggesting hyperplasia to be a cause or consequence of aneurysm formation (Fig. 3G,H). Furthermore, the medial fraction of collagen correlated most closely to adventitia thickness, suggesting a common stimulus for vessel wall fibrosis (Fig. 3I). These findings imply diverse interactions among medial components and among vessel wall layers in response to mechanical stresses and biological stimuli arising during aneurysm development.

### Age and bicuspid aortic valve influence medial elastin expression and SMC number

Since the subjects with nondilated and aneurysmal aortas differed for age, an important risk factor of aortic disease, we repeated comparisons after excluding subjects ≤ 40 years old. Aneurysms contained increased SMC but similar elastin to nondilated aortas among older subjects, thus obscuring the decreased medial fraction of elastin seen with subjects of all ages (Supplementary Table S3). A comparison of young versus older subjects with only nondilated aortas confirmed decreased medial fraction of elastin and SMC density with ageing, although cross-sectional elastin presence and total number of SMC per cross-section were maintained because of increased media area (Supplementary Table S4 and Supplementary Fig. S1). Thus, comparison of aneurysmal aortas with nondilated aortas of younger subjects will underestimate the degree of SMC hyperplasia and overstate any loss/dilution of elastin. As the study groups also differed for the incidence of bicuspid aortic valve, another determinant of aortic pathology, we performed a further subgroup analysis in aneurysmal aortas with bicuspid or tricuspid aortic valves. The number of aortic valve leaflets did not associate with differences in media thickness or SMC number, but medial fraction of elastin was lower in aortas with normal valve morphology (Supplementary Table S5). Hence, the finding of only moderately decreased medial fraction of elastin in aortic aneurysms may have been skewed by including subjects with bicuspid aortic valves. These results highlight potential confounders when clinical characteristics of aorta subjects are not matched between groups.

### Less than expected thinning of elastic lamellae and narrowing of interlamellar distance suggest net gain of medial components in aortic aneurysm remodeling

**Figure 4:**
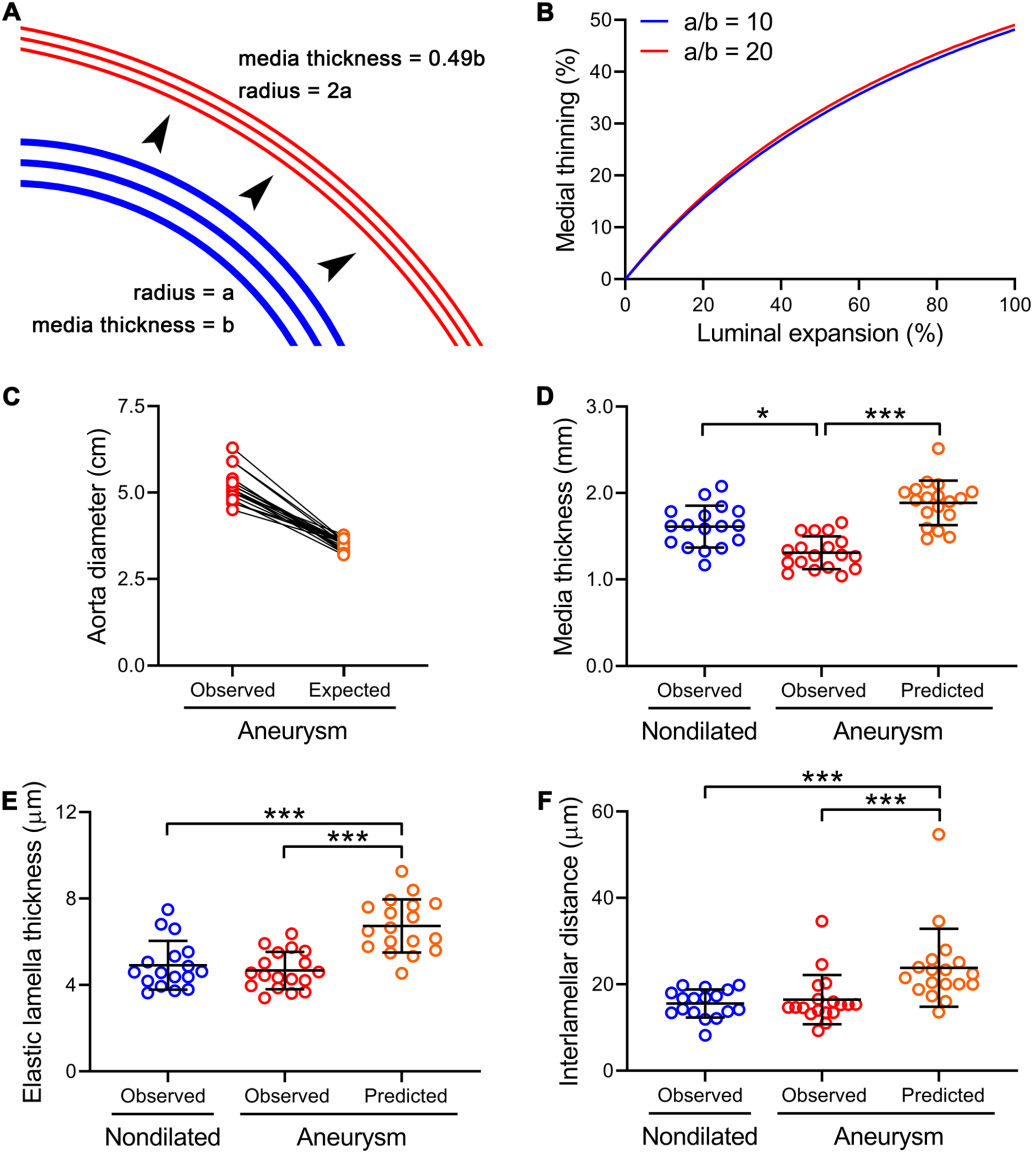
Gain of medial components in aortic aneurysms. (**A**) Enlargement of the lumen is associated with thinning of the media and its concentric lamellae assuming preservation of medial area and (**B**) this relationship is described graphically for radius (a) to medial thickness (b) indices of 10 (typical of nondilated aortas) and 20 (typical of aneurysmal aortas). The derived equation (“Supplementary Appendix”) was applied to quantitative histomorphometry data of ascending aorta specimens from organ donors and patients undergoing aneurysm repair. (**C**) Luminal enlargement was calculated from observed to expected aortic diameters of aneurysm patients according to published nomograms of healthy subjects. (**D**) Media thickness, (**E**) elastic lamella thickness, and (**F**) interlamellar distance of nondilated aortas and observed and predicted values for aneurysmal aortas. Individual data shown with bars representing mean ± SD, *n* = 17 nondilated and *n* = 18 aneurysms, *P < 0.05, ***P < 0.001 aneurysm versus nondilated, Kruskal–Wallis test with Dunn’s multiple comparisons test.

We further examined if the quantitative histomorphometry findings fit with net degradative versus synthetic remodeling of the media among the subgroups with aneurysmal versus nondilated aortas. A media consisting of concentric lamellae will thin proportional to enlargement of the lumen if there is no change in material composition via a relationship that can be described mathematically (Fig. 4A,B and “Supplementary Appendix”). If the media thins more than predicted, then medial area has decreased due to ECM degradation and/or SMC death. Conversely, if the media thins less than predicted (or thickens), then medial area has increased due to ECM synthesis and/or SMC hyperplasia/hypertrophy. To calculate the degree of luminal enlargement, the expected aorta diameter for each aneurysm patient was derived from age-, sex-, and body surface area-specific nomograms of healthy subjects^12^ and aorta size differed 1.5-fold between expected (3.5 ± 0.2 cm) and observed (5.2 ± 0.5 cm) diameters (Fig. 4C). Applying the expected change in lumen size to the equation derived in the appendix predicted pre-aneurysmal media thickness, elastic lamella thickness, and interlamellar distance to be larger than that measured in nondilated aortas if vessel wall components had remained inert (Fig. 4D–F). Although it is possible that patients with bicuspid aortic valves have developmental changes in vascular morphology, the similar hypothetical reverse remodeling results for aortas with normal valves suggest a gain of medial components during aortic aneurysm formation that partially compensates for medial thinning resulting from passive stretching (Supplementary Fig. S2).

## Discussion

We find that qualitative histopathology, when performed in blinded fashion on tissue specimens of standardized location and orientation, does not differentiate sporadic thoracic aortic aneurysms from nondilated aortas because of significant overlap in abnormalities. Medial degeneration has long been described in nondilated aortas of older individuals^2,13^. Our findings identified degenerative changes in aneurysms commonly associated with ageing, thus underscoring the importance of appropriate age-matched controls in studies of aortic disease. Even including a subset of younger subjects with mild medial degeneration, the control aortas herein could not be differentiated from aneurysms. Importantly, sporadic aneurysms tend to manifest less severe medial degeneration than certain syndromic aneurysms, such as those in Marfan syndrome, and aneurysms associated with congenital bicuspid aortic valve have less medial degeneration than sporadic aneurysms^14^. Our results partially reflect this spread of medial abnormalities. Nevertheless, quantitative measures discriminated between aortic aneurysms and referent specimens despite marked overlap in results. The common focus on more abnormal fields during standard histopathologic examination may explain the discrepancy with histomorphometrical assessment of multiple representative fields in all parts of the media. Examination for localized defects is important, however, as these may cause lesions to grow more rapidly or have a greater risk of complication than lesions with uniform degeneration.

An important question in aortic aneurysm pathogenesis is whether medial degeneration is cause or effect, and if the former, is it sufficient or necessary for disease? Although descriptive human studies cannot ascribe causality to possible mechanisms, qualitatively indistinguishable grades of medial degeneration in aneurysmal versus nondilated aortas argues against sufficiency. Conversely, the frequent occurrence of medial degeneration in aortic aneurysms suggests necessity. Previous studies have found as few as 0 of 339 to as many as 90 of 514 ascending aortic aneurysms without histological abnormalities, though definitions of disease varied and aortic fragments were likely randomly selected for histopathological examination^4,15^. The near ubiquitous presence of medial degeneration in aortic aneurysms may also reflect secondary effects of initially greater stresses on the dilated vessel wall. The relatively small aortic aneurysms (mean diameter 5.2 cm) we studied may have been subjected to lower deviations in wall stress from normal and developed fewer secondary abnormalities unlike pathogenic mechanisms that are evident early in the disease course. However, experimental models of ascending aortic aneurysms universally exhibit medial degeneration during aneurysm formation suggesting a causative role at least for genetic, chemical, and physical stimuli used in these systems^6^. Regardless of a primary or secondary phenomenon in sporadic aortic aneurysms, we speculate that disorganization of elastic fibers and SMC leads to altered mechanotransduction, which exaggerates maladaptive remodeling of the vessel wall^16^.

In contradistinction to the theory that SMC death causes aneurysm pathogenesis, we did not observe loss of SMC in these sporadic ascending aorta aneurysms; rather, their total number per cross-section increased consistent with the greater cross-sectional expression of cytoplasm in the media and preservation of interlamellar distance despite stretching of the vessel wall. We have previously described unchanged SMC density and increased total number in a separate cohort of 57 subjects, though without standardizing tissue location and orientation or quantifying the parameters in blinded fashion^17^. Counting medial nuclei on H&E stains overestimates the number of SMC as vasa vasorum endothelial cells and infiltrating leukocytes are also counted, although Ulex+ and CD45+ cells are more than an order of magnitude less than smooth muscle α-actin+ cells within the media of nondilated and aneurysmal ascending aortas^17,18^. Other investigators have reported unchanged, increased, or decreased SMC density in ascending aorta aneurysms, though cross-sectional data were not calculated^19–21^. Furthermore, in qualitative comparisons of syndromic and sporadic aneurysms without control nondilated aortas, SMC loss was uncommon in Marfan and Loeys-Dietz syndromes but instead was a feature of ageing^14^. Consistent with a hypothesis for increased turnover of the ECM in aortic aneurysms, death of SMC may lessen further dilatation of fibrotic tissue and represent burnout of disease unless the media is infiltrated by other cell types capable of ECM remodeling, such as leukocytes.

Our observations of changes in elastin are complex. The medial fraction of elastin is decreased likely from dilution as cross-sectional amounts of protein are maintained. On the other hand, elastic lamellae thinned less than expected with stretching of the vessel wall suggesting active synthesis. This conclusion may not appear to be in agreement with the paradigm that functional elastin synthesis is completed early in postnatal development^22^. Those findings, though, were in physiological settings and it appears that limited elastin production occurs in different vascular disease states^23,24^, though this produced elastin probably does not confer normal mechanical properties, mainly elastic energy storage capability^25,26^. Indeed, elastin synthesis assessed by mRNA expression has been detected in ascending aortic aneurysms^17^. An alternative explanation is that enlargement of lamellar fenestrations or rupture of elastic lamellae prevents thinning commensurate with stretching of the aneurysm wall. A similar effect was not seen, however, in ageing aortas with similar grades of elastin fragmentation. Nonetheless, decreased undulation, altered direction, and disruption of elastic fibers is evident in ascending aortic aneurysms^27^. The observation that elastic lamellae appeared to thicken whereas cross-sectional elastin was not increased may indicate selective loss of elastin associated with microfibrils and lamellar extensions. Up to 29% of medial elastin could be intralamellar^28^, with many of these fibers contributing to mechano-sensing^29^. Two of the aneurysm specimens we examined were low outliers for cross-sectional elastin consistent with marked loss of elastic fibers; the patients’ co-morbidities were unremarkable and without a family history of aortic aneurysms. Our results in sporadic aneurysms of the ascending aorta are likely not applicable to syndromic conditions associated with primary defects of ECM molecules, such as Marfan syndrome.

Our strategy of deriving cross-sectional parameters from focal wall biopsies has several caveats, including assumptions of axisymmetry. Although curvature of the ascending aorta does not impact our calculations at a particular transverse plane and consistent measurements at a specific location minimizes variation if thickness differs along the circumference, mechanical stresses and medial abnormalities can vary with circumferential location of the ascending aorta^30,31^. We studied the anterior greater curvature of the mid-ascending aorta where sporadic aneurysms are most common and severe and thus are unlikely to be sampling less diseased locations. By not analyzing random specimen fragments (in which the aneurysm neck may be selected instead of the belly), we did not analyze replicates to minimize sampling variation^15^. Since our cross-sectional calculations are 2-dimensional, we may underestimate a greater increase in medial volume and stretching of elastic lamellae as the ascending aorta is known to lengthen with aneurysmal disease^32^. Attempting to cut and microscopically analyze complete aortic rings up to 6 cm in size is not practical and biochemical assessment of protein is challenging due to the relatively insoluble nature of the ECM and moreover would not discriminate expression within different vessel wall layers.

In conclusion, our findings are not in disagreement with previous studies that have documented elastic fiber fragmentation or degradation and SMC death in ascending aortic aneurysms, but extend those observations in that concomitant elastin synthesis and SMC proliferation can lead to a net gain of medial tissue and an increase in cross-sectional area of the media in a significant subset of sporadic disease. While standard histology is informative, it does not recognize material turnover suggested by our findings of elastic lamella thickening and SMC hyperplasia. Our interpretation is that continual remodeling of the media during aortic aneurysm enlargement is not perfectly coordinated, resulting in disorganization of elastic fibers and SMC as the defining characteristics of medial degeneration rather than perceived loss of individual components.

## Methods

### Aorta specimens

Transverse rectangular tissue samples from the right anterolateral (greater curvature), mid-ascending aorta were obtained from patients undergoing aortic aneurysm repair surgery (without or with concomitant aortic valve surgery) or from organ donors with nondilated aortas whose hearts were not used for clinical transplantation. The intrapericardial location of this part of the aorta means that dissection from contiguous tissues is not required and specimens represent full-thickness aortic wall. The specimens were procured by the investigators within the operating room to ensure precise anatomical location and orientation. The samples were not obtained from consecutive cases at the institutions but represented all cases during the study period that the investigators were available to process the tissues. Demographic and clinical information was obtained from the electronic records. None of the subjects had known genetic conditions predisposing to aortic aneurysms. The specimens were coded without patient identifiers. Aorta diameters were based on preoperative radiological studies and when not available by direct measurement in vivo. Expected aorta diameters were calculated from linear regression equations of age-, sex-, and body surface area-specific nomograms for healthy subjects^12^ using a custom calculator (https://medicine.yale.edu/surgery/cardio/research/) suitable for individual subjects or large databases that was recently described^33^. Aortic aneurysms are defined as > 1.5 times the expected aorta diameter, while aortas within 2 SD of populations means are considered normal^34,35^.

### Histological stains

Aortic tissue was fixed in 4% paraformaldehyde overnight and embedded in paraffin. Serial 5 μm-thick transverse sections were stained in single batches by Yale's Research Histology Laboratory. H&E, VVG, Verhoeff without counterstain, Masson’s trichrome, and Movat’s pentachrome stains were performed using standard techniques and an automated system.

### Histopathology

Three pathologists with varying levels of training (attending, fellow, and resident) independently reviewed the histological slides blinded to the clinical diagnosis. The severity and extent of medial degeneration was scored based on the consensus statement from the Society for Cardiovascular Pathology and the Association for European Cardiovascular Pathology on nomenclature and diagnostic criteria for noninflammatory degenerative aortic diseases^3^. The categories, grade, and distribution of medial degeneration are listed in Table 2 and Supplementary Table S1 according to diagnostic criteria detailed and illustrated in the aforementioned consensus statement.

### Histomorphometry

Morphometric measurements were performed by three investigators blinded to the clinical diagnosis. Digital images were obtained using an Axioskop 2 microscope with KS 300 Imaging software (Carl Zeiss). Medial fraction (percent area of positive staining) of elastin, cytoplasm, collagen, and glycosaminoglycans were measured from Movat stains using a custom color segmentation algorithm for MATLAB with Statistics Toolbox (MathWorks). Medial fraction of elastin was also measured from Verhoeff stains using ImageJ software (http://rsbweb.nih.gov/ij/). The medial constituents were measured from 9 arbitrarily selected fields (3 each of the inner, mid, and outer media) and averaged. Vessel layer thickness was measured from VVG stains using ImageJ software and averaged from 3 sites. The number of SMC was calculated from H&E stains by averaging counts of medial nuclei from 9 fields. Elastic lamellae thickness and interlamellar distance were measured from Verhoeff stains and averaged from 9 replicates. Since elastic lamellae vary in thickness, in particular thinning after dividing into two lamellae or after takeoff of radial elastin struts that join adjacent lamellae, we measured lamellar thickness either in segments without divisions or immediately prior to branches and struts (Supplementary Fig. S3). The number of lamellae were counted from VVG stains using high quality digital images with Aperio eSlide Manager (Leica Biosystems).

### Statistics

Quantitative data are presented as dot plots of individual values with overlying bars representing mean and SD. Nonparametric tests were used because of the relatively low number of subjects (i.e., < 30 per group). Comparison of continuous variables between 2 groups was by Mann–Whitney test and between more than 2 groups by Kruskal–Wallis test followed by Dunn’s multiple comparisons tests if the null hypothesis was rejected (Prism 8.2.0, GraphPad Software). Comparison of categorical variables was by Fisher’s exact test (SAS 9.4, SAS Institute). Inter-rater reliability for overall medial degeneration score was by Spearman’s rank correlation coefficient as this was an ordinal variable ranging 1 to 3 (SAS 9.4, SAS Institute). Correlation of quantitative histology data were examined by Spearman correlation (Prism 8.2.0, GraphPad Software). Probability values were 2-tailed and *P* < 0.05 was considered to indicate statistical significance.

### Study approval

Human subjects research protocols with a waiver for consent were approved by the Human Research Protection Program of Yale University (protocol ID 2000020632), the Research and Development Committee of the Veterans Affairs Connecticut Healthcare System (MIRB 00151), and the Research Review Committee of New England Donor Services. All analyses were performed in accordance with relevant guidelines and regulations.

## Supplementary Information

Supplementary Information.

## Data Availability

The datasets generated during and/or analyzed during the current study are available from the corresponding author on reasonable request.
