# Supplementary material for: Quantitative not qualitative histology differentiates aneurysmal from nondilated ascending aortas and reveals a net gain of medial components
Source: Sci Rep. 2021 Jun 23;11:13185. doi: 10.1038/s41598-021-92659-1 (PMC8222259; doi:10.1038/s41598-021-92659-1)
Supplement: Supplementary file 1 — Supplementary Information. [file 41598_2021_92659_MOESM1_ESM.pdf]

Supplementary Materials

Quantitative not Qualitative Histology Differentiates Aneurysmal from Nondilated Ascending Aortas and Reveals a Net Gain of Medial Components

Sameh Yousef[1], Nana Matsumoto[2], Issam Dabe[1], Makoto Mori[1], Alden B. Landry[1], Shin-Rong Lee[1], Yuki Kawamura[1], Chen Yang[2], Guangxin Li[1,3], Roland Assi[1,4,5], Prashanth Vallabhajosyula[1], Arnar Geirsson[1,4], Gilbert Moeckel[2], Jay D. Humphrey[4,6], George Tellides[1,4,5,*]

[1]Section of Cardiac Surgery, Department of Surgery, Yale School of Medicine, New Haven, CT.
[2]Department of Pathology, Yale School of Medicine, New Haven, CT, USA.
[3]Department of Breast and Thyroid Surgery, Peking University Shenzhen Hospital, Shenzhen, Guangdong Province, China.
[4]Vascular Biology and Therapeutics Program, Yale School of Medicine, New Haven, CT, USA.
[5]Veterans Affairs Connecticut Healthcare System, West Haven, CT, USA.
[6]Department of Biomedical Engineering, Yale School of Engineering and Applied Science, New Haven, CT, USA.

*Correspondence: George Tellides, 10 Amistad Street 337B, New Haven, CT 06520, USA. Phone: 203-737-2298; Fax: 203-737-6386; E-mail: george.tellides@yale.edu

Supplementary Appendix: Changes in Media Thickness with Aortic Aneurysm Formation

The relationship of media thickness to changes in lumen size assuming preservation of the cross-sectional area of the media can be described by a simple mathematical study. Let the normal luminal radius be denoted by a and the normal medial thickness be denoted by b. Medial cross-sectional area A is thus given by $A = \pi b(2a + b)$. If we let a perturbed luminal radius be given by αa and the associated perturbed medial thickness be given by βb, where α and β simply denote fold-changes (e.g., $\alpha = 1.5$ if the inner radius increases by 50%) and if we assume that the perturbed (new) cross-sectional area equals the normal area, then $A = \pi b(2a + b) = \pi\beta b(2\alpha a + \beta b)$, which admits a solution for the change in medial thickness that is required to maintain medial area constant despite a change in luminal radius, namely

$$\beta = -\alpha\left(\frac{a}{b}\right) \pm \sqrt{\alpha^2\left(\frac{a}{b}\right)^2 + 2\left(\frac{a}{b}\right) + 1}. \qquad (1)$$

Consequences of this constraint are seen in Fig. 4B. If the media thickness is less than predicted, then loss of media area due to degradation of ECM and/or SMC death can be inferred. Conversely, if the media thickness is greater than predicted, then gain of media area due to synthesis of ECM and/or SMC hypertrophy/hyperplasia can be inferred.

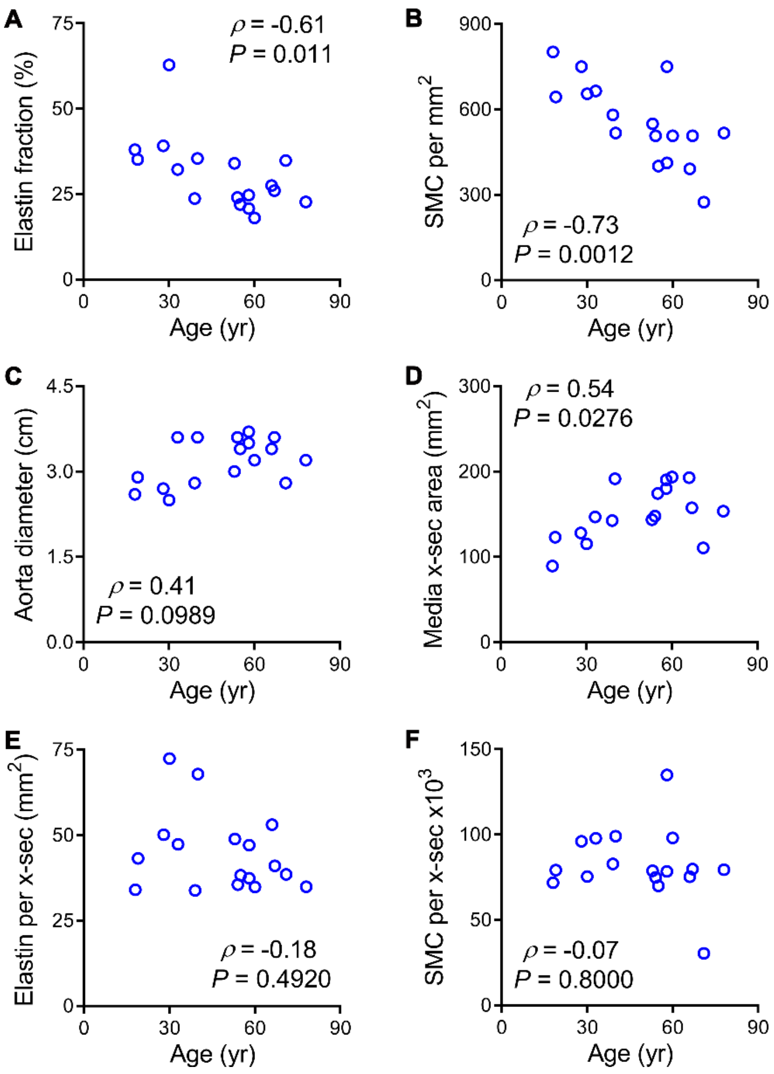

Supplementary Fig. S1: Correlation of medial fraction of elastin and SMC density with age. Nondilated ascending aortas from organ donors were analyzed by histomorphometry. Correlation of age to (***A***) medial fraction of elastin from Movat stain, (***B***) number of SMC per mm^2, (***C***) aorta diameter, (***D***) medial cross-sectional (x-sec) area, (***E***) cross-sectional area of elastin, and (***F***) number of SMC per cross-section. Individual data shown, *n* = 17, and comparisons were by Spearman correlation.

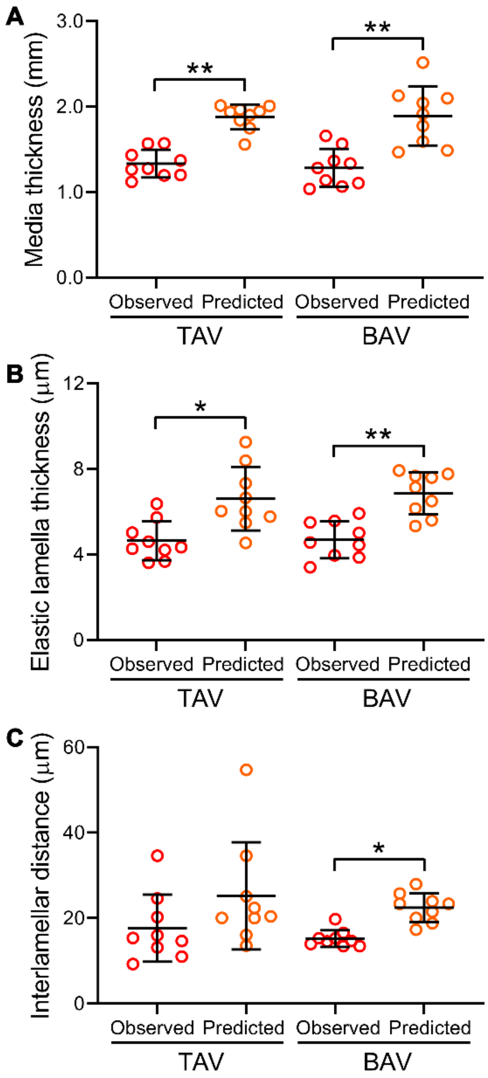

Supplementary Fig. S2: Similar hypothetical reverse remodeling of aortic aneurysms with tricuspid and bicuspid aortic valves. Observed and predicted (***A***) media thickness, (***B***) elastic lamella thickness, and (***C***) interlamellar distance of aneurysmal aortas with tricuspid (TAV) or bicuspid (BAV) aortic valves. Individual data shown with bars representing mean ± SD, TAV n = 9 and BAV n = 9, *$P < 0.05$, **$P < 0.01$ predicted versus observed, comparisons were by Kruskal-Wallis test with Dunn's multiple comparisons test.

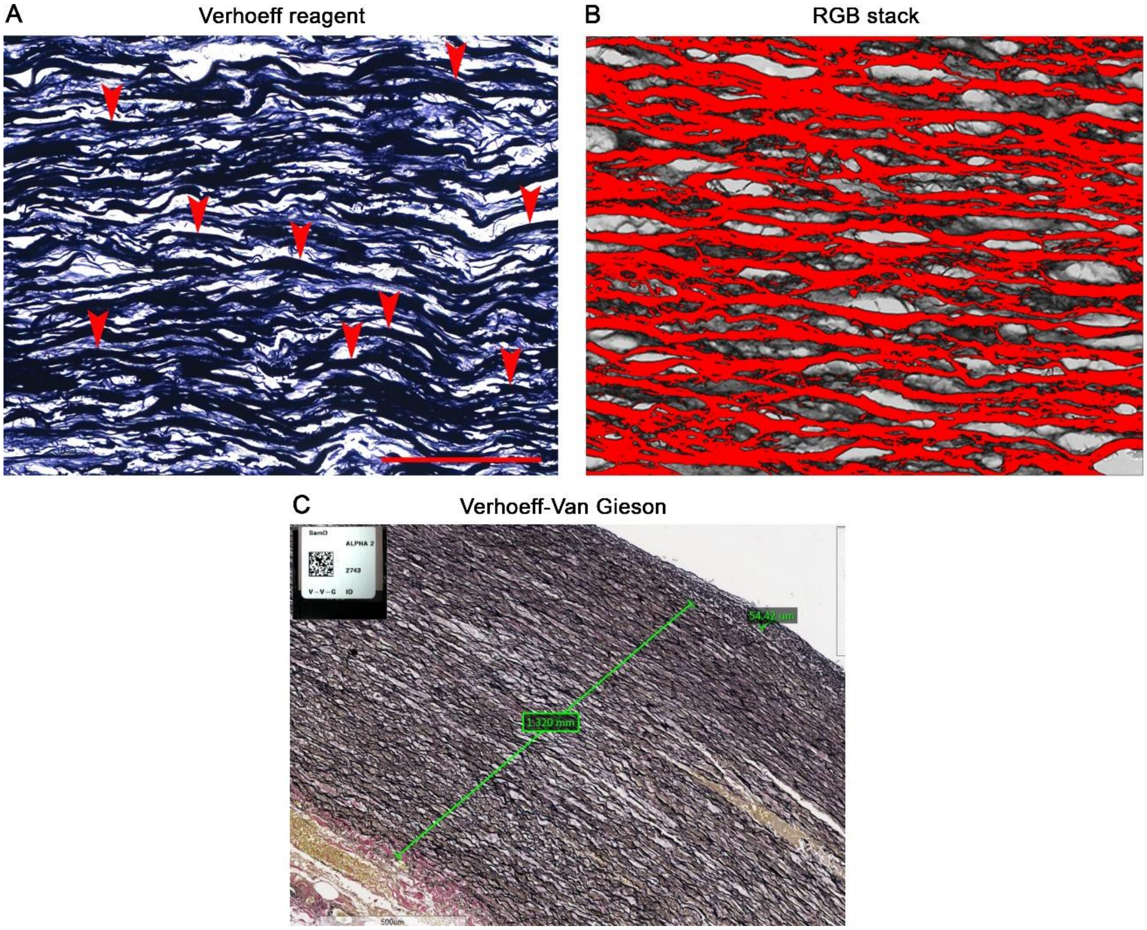

Supplementary Fig. S3: Quantification of elastic fibers. (***A***) Elastic lamellar thickness and interlamellar distance were measured at high magnification of sections stained with Verhoeff reagent alone (without Van Gieson counterstain). Since the thickness of an elastic lamella is not uniform, often thinning after branching into two or after the takeoff of a strut joining adjacent lamellae, we measured thickness at 9 sites per section either in segments without divisions or immediately prior to branching or struts (red arrows). Scale bar represents 100 µm. (***B***) Elastic fraction of media was measured using ImageJ (1. File > Open, 2. Image > Type > RGB stack, 3. Image > Adjust > Threshold, 4. Analyze > Set Measurement, and 5. Analyze > Measure) with the intended area measured turning a red color and quantified as area fraction which is the ratio of red colored area to total area. The pseudo-colored image shows branching and struts of elastic lamellae more clearly than the original. Most of the minor interlamellar elastic fibers with a grey appearance are not pseudo-colored red and thus are not included in the fraction of more substantial elastic lamellae and elastin struts. (***C***) The number of lamellae crossing a line from internal to external elastic laminae were counted in a single, high quality digital image.

Supplementary Table S1: Qualitative blinded scoring of medial degeneration*

		Observer 2			Observer 3		
		Nondilated *n* = 17	Aneurysm *n* = 18	*P* value	Nondilated *n* = 17	Aneurysm *n* = 18	*P* value
Medial degeneration	Mild	6 (35%)	2 (11%)	0.2185	6 (35%)	2 (11%)	0.2684
	Moderate	4 (24%)	8 (44%)		6 (35%)	8 (44%)	
	Severe	7 (41%)	8 (44%)		5 (29%)	8 (44%)	
MEMA-Intralamellar grade	None	1 (6%)	0 (0%)	>0.99	0 (0%)	0 (0%)	0.7270
	Mild	9 (53%)	10 (56%)		10 (59%)	8 (44%)	
	Moderate	4 (24%)	5 (28%)		5 (29%)	7 (39%)	
	Severe	3 (18%)	3 (17)		2(12%)	3(17%)	
MEMA-Intralamellar distribution	Absent	1 (6%)	0 (0%)	0.2895	0 (0%)	0 (0%)	0.0804
	Focal	5 (29%)	2 (11%)		4 (24%)	2 (11%)	
	Multifocal	5 (29%)	5 (28%)		9 (53%)	5 (28%)	
	Extensive	6 (35%)	11 (61%)		4 (24%)	11 (61%)	
MEMA-Translamellar grade	None	14 (82%)	9 (50%)	0.1919	10 (59%)	6 (33%)	0.2771
	Mild	1 (6%)	4 (22%)		5 (29%)	6 (33%)	
	Moderate	1 (6%)	4 (22%)		1 (6%)	5 (28%)	
	Severe	1 (6%)	1 (6%)		1 (6%)	1 (6%)	
MEMA-Translamellar distribution	Absent	14 (82%)	9 (50%)	0.0793	10 (59%)	6 (33%)	0.0764
	Focal	0 (0%)	3 (17%)		4 (24%)	11 (61%)	
	Multifocal	3 (18%)	6 (33%)		3 (18%)	1 (6%)	
EFFL grade	None	5 (29%)	5 (28%)	0.6069	6 (35%)	4 (22%)	0.8137
	Mild	9 (53%)	7 (39%)		8 (47%)	9 (50%)	
	Moderate	1 (6%)	4 (22%)		1 (6%)	3 (17%)	
	Severe	2 (12%)	2 (11%)		2 (12%)	2 (11%)	
EFFL distribution	Absent	5 (29%)	5 (28%)	0.9526	6 (35%)	4 (22%)	0.7757
	Focal	6 (35%)	5 (28%)		6 (35%)	6 (33%)	
	Multifocal	5 (29%)	7 (39%)		4 (24%)	7 (39%)	
	Extensive	1 (6%)	1 (6%)		1 (6%)	1 (6%)	
EFT grade	None	10 (59%)	10 (56%)	>0.99	9 (53%)	9 (50%)	>0.99
	Mild	4 (24%)	5 (28%)		5 (29%)	6 (33%)	
	Moderate	1 (6%)	2 (11%)		1 (6%)	2 (11%)	
	Severe	2 (12%)	1 (6%)		2 (12%)	1 (6%)	
EFT distribution	Absent	10 (59%)	10 (56%)	0.4418	9 (53%)	9 (50%)	>0.99
	Focal	3 (18%)	3 (17%)		4 (24%)	5 (28%)	
	Multifocal	1 (6%)	4 (22%)		2 (12%)	3 (17%)	
	Extensive	3 (18%)	1 (6%)		2 (12%)	1 (6%)	
EFD distribution	Absent	4 (24%)	3 (17%)	>0.99	3 (18%)	2 (11%)	0.8932
	Focal	6 (35%)	6 (33%)		8 (47%)	8 (44%)	
	Multifocal	4 (24%)	5 (28%)		4 (24%)	4 (22%)	
	Extensive	3 (18%)	4 (22%)		2 (12%)	4 (22%)	
SMCNL type	None	5 (29%)	5 (28%)	0.8825	2 (12%)	2 (11%)	0.8625
	Patchy	10 (59%)	12 (61%)		12 (71%)	14 (78%)	
	Band-like	2 (12%)	1 (6%)		3 (18%)	2 (11%)	
SMCNL distribution	Absent	5 (29%)	5 (28%)	>0.99	2 (12%)	2 (11%)	0.0546
	Rare	10 (59%)	11 (67%)		11 (65%)	6 (33%)	
	Frequent	0 (0%)	1 (6%)		1 (6%)	8 (44%)	
	Extensive	2 (12%)	1(6%)		3 (18%)	2 (11%)	
LMC appearance	None	13 (76%)	9 (50%)	0.2058	12 (71%)	6 (33%)	0.0466
	Thin	3 (18%)	8 (44%)		4 (24%)	11 (61%)	
	Dense	1 (6%)	1 (6%)		1 (6%)	1 (6%)	
LMC distribution	Absent	13 (76%)	9 (50%)	0.0355	12 (71%)	6 (33%)	0.0094
	Focal	2 (12%)	8 (44%)		2 (12%)	10 (56%)	
	Multifocal	0 (0%)	1 (6%)		1 (6%)	2 (11%)	
	Extensive	2 (12%)	0 (0%)		2 (12%)	0 (0%)	
SMCD distribution	Absent	12 (71%)	12 (67%)	>0.99	8 (47%)	10 (56%)	0.8543
	Focal	2 (12%)	1 (6%)		4 (24%)	2 (11%)	
	Multifocal	1 (6%)	2 (11%)		3 (18%)	3 (17%)	
	Extensive	2 (12%)	3 (17%)		2 (12%)	3 (17%)	

Medial fibrosis grade	None	2 (12%)	1 (6%)	0.5845	2 (12%)	1 (6%)	0.4902
	Mild	11 (65%)	15 (83%)		10 (59%)	15 (83%)	
	Moderate	2 (12%)	1 (6%)		3 (18%)	1 (6%)	
	Severe	2 (12%)	1 (6%)		2 (12%)	1 (6%)	
Medial fibrosis distribution	Absent	2 (12%)	1 (28%)	0.9212	2 (12%)	1 (6%)	0.8821
	Focal	8 (47%)	8 (44%)		7 (41%)	9 (50%)	
	Multifocal	3 (18%)	5 (28%)		5 (29%)	6 (33%)	
	Extensive	4 (24%)	4 (22%)		3 (18%)	2 (11%)	

*Qualitative (semiquantitative) scoring of medial degeneration by 2 additional pathologists of ascending aorta specimens from organ donors (nondilated) and patients undergoing aneurysm repair (aneurysm). Histopathology was assessed blinded to the clinical diagnosis. Diagnostic criteria include overall medial degeneration score and several individual components of medial degeneration categorized by grade and distribution (subcategories without scores are not shown). Severe medial fibrosis was translamellar, whereas all lesser grades of medial fibrosis were intralamellar. The categorical variables are represented as numbers and percentage of total. Comparisons of criteria with multiple subcategory scores between groups are by Fisher's exact test. MEMA: mucoid ECM accumulation, EFFL: elastic fiber fragmentation and/or loss, EFT: elastic fiber thinning, EFD: elastic fiber disorganization, SMCNL: SMC nuclei loss, LMC: laminar medial collapse, SMCD: SMC disorganization.

**ρ coefficients**	Aorta diameter	ELN fraction Movat	CYT fraction Movat	COL fraction Movat	GAG fraction Movat	ELN fraction Verhoeff	Lamellae number	Elastic lamella thickness	Interlamellar distance	SMC per mm^2	Intima thickness	Media thickness	Adventitia thickness	Media x-sec area	SMC per x-sec
Aorta diameter	1	-0.44	0.33	-0.05	0.27	-0.35	-0.23	-0.13	0.08	-0.01	0.10	-0.54	-0.29	0.77	0.72
ELN fraction Movat	-0.44	1	-0.70	-0.31	-0.30	0.61	0.31	0.33	-0.40	-0.05	-0.28	0.13	-0.27	-0.39	-0.46
CYT fraction Movat	0.33	-0.70	1	0.66	-0.31	-0.53	-0.13	-0.32	0.21	0.12	0.18	-0.10	0.32	0.29	0.38
COL fraction Movat	0.05	-0.31	0.66	1	-0.67	-0.31	-0.06	-0.25	0.04	-0.23	0.01	0.06	0.40	0.10	-0.04
GAG fraction Movat	0.27	-0.30	-0.31	-0.67	1	-0.00	-0.26	0.06	0.11	0.04	0.16	-0.09	-0.30	0.22	0.28
ELN fraction Verhoeff	-0.35	0.61	-0.53	-0.31	-0.00	1	0.20	0.67	-0.45	-0.05	-0.28	0.01	-0.18	-0.41	-0.35
Lamellae number	-0.23	0.31	-0.13	-0.06	-0.26	0.16	1	-0.11	-0.14	-0.19	-0.11	0.25	-0.04	-0.09	-0.20
Elastic lamella thickness	-0.13	0.33	-0.32	-0.25	0.06	0.67	-0.11	1	-0.41	-0.11	-0.13	-0.13	-0.19	-0.14	-0.14
Interlamellar distance	0.08	-0.40	0.21	0.04	0.11	-0.45	-0.14	-0.41	1	0.00	0.41	0.20	0.27	0.19	0.11
SMC per mm^2	-0.01	-0.05	0.12	-0.23	0.04	-0.05	-0.19	-0.11	0.00	1	0.01	-0.25	0.21	-0.10	0.40
Intima thickness	0.10	-0.28	0.18	0.01	0.16	-0.28	-0.11	-0.13	0.41	0.01	1	-0.04	0.12	0.10	0.04
Media thickness	-0.54	0.13	-0.10	0.06	-0.09	0.01	0.25	-0.13	0.20	-0.25	-0.04	1	0.26	0.02	-0.15
Adventitia thickness	-0.29	-0.27	0.32	0.40	-0.30	-0.18	-0.04	-0.19	0.27	0.21	0.12	0.26	1	-0.10	-0.12
Media x-sec area	0.77	-0.39	0.29	0.10	0.22	-0.41	-0.09	-0.14	0.19	-0.10	0.10	0.02	-0.10	1	0.79
SMC per x-sec	0.72	-0.46	0.38	-0.04	0.28	-0.35	-0.20	-0.14	0.11	0.40	0.04	-0.15	-0.12	0.79	1
***P* values**															
Aorta diameter		0.0076	0.0547	0.7677	0.1124	0.0376	0.1922	0.4517	0.6486	0.9502	0.5779	0.0008	0.0967	6.4E-08	1.3E-06
ELN fraction Movat	0.0076		2.7E-06	0.0698	0.0842	0.0001	0.0730	0.0566	0.0171	0.7726	0.1047	0.4696	0.1168	0.0209	0.0059
CYT fraction Movat	0.0547	2.7E-06		1.5E-05	0.0734	0.0011	0.4591	0.0617	0.2164	0.5031	0.2993	0.5639	0.0583	0.0870	0.0253
COL fraction Movat	0.7677	0.0698	1.5E-05		1.1E-05	0.0743	0.7256	0.1428	0.8405	0.1920	0.9662	0.7326	0.0172	0.5681	0.8255
GAG fraction Movat	0.1124	0.0842	0.0734	1.1E-05		0.9873	0.1289	0.7263	0.5396	0.8065	0.3620	0.5887	0.0768	0.1974	0.1072
ELN fraction Verhoeff	0.0376	0.0001	0.0011	0.0743	0.9873		0.3480	1.2E-05	0.0061	0.7763	0.0980	0.9509	0.3132	0.0135	0.0368
Lamellae number	0.1922	0.0730	0.4591	0.7256	0.1289	0.3480		0.5222	0.4383	0.2773	0.5454	0.1488	0.8265	0.5908	0.2447
Elastic lamella thickness	0.4517	0.0566	0.0617	0.1428	0.7263	1.2E-05	0.5222		0.0155	0.5255	0.4641	0.4686	0.2828	0.4102	0.4084
Interlamellar distance	0.6486	0.0171	0.2164	0.8405	0.5396	0.0061	0.4383	0.0155		0.9802	0.0148	0.2553	0.1103	0.2855	0.5279
SMC per mm^2	0.9502	0.7726	0.5031	0.1920	0.8065	0.7763	0.2773	0.5255	0.9802		0.9490	0.1498	0.2340	0.5598	0.0173
Intima thickness	0.5779	0.1047	0.2993	0.9662	0.3620	0.0980	0.5454	0.4641	0.0148	0.9490		0.8029	0.4804	0.5659	0.8193
Media thickness	0.0008	0.4696	0.5639	0.7326	0.5887	0.9509	0.1488	0.4686	0.2533	0.1498	0.8029		0.1342	0.9134	0.3848
Adventitia thickness	0.0967	0.1168	0.0583	0.0172	0.0768	0.3132	0.8265	0.2828	0.1103	0.2340	0.4804	0.1342		0.5588	0.4911
Media area	6.4E-08	0.0209	0.0870	0.5681	0.1974	0.0135	0.5908	0.4102	0.2855	0.5598	0.5659	0.9134	0.5588		1.6E-08
SMC per x-sec	1.3E-06	0.0059	0.0253	0.8255	0.1072	0.0368	0.2447	0.4084	0.5279	0.0173	0.8193	0.3848	0.4911	1.6E-08	

Supplementary Table S2: Correlation matrix. Ascending aorta specimens from organ donors and patients undergoing aneurysm repair were analyzed by histomorphometry. Correlations were determined among continuous variables for both nondilated and aneurysm specimens (n = 35). Upper matrix shows Spearman correlation coefficients and the lower matrix shows the corresponding P values. ELN: elastin, CYT: cytoplasm, COL: collagen, GAG: glycosaminoglycans, x-sec: cross-section.

Supplementary Table S3: Influence of age on histomorphometry parameters in nondilated and aneurysmal aortas*

	Nondilated > 40 yr<br>*n* = 10	Aneurysm > 40 yr<br>*n* = 18	*P* value
Age	62.0 ± 8.2	62.7 ± 11.0	0.8781
Aorta diameter (cm)	3.34 ± 0.29	5.19 ± 0.46	<0.0001
Aorta z-score	-0.13 ± 0.86	5.56 ± 1.77	<0.0001
Intima thickness (mm)	0.22 ± 0.44	0.21 ± 0.36	0.6457
Media thickness (mm)	1.67 ± 0.25	1.31 ± 0.19	0.0004
Adventitia thickness (mm)	0.15 ± 0.14	0.10 ± 0.06	0.1946
Media x-sec area (mm^2)	164 ± 27	207 ± 30	0.0009
SMC per mm^2	481 ± 126	577 ± 64	0.0110
SMC per x-sec $x10^3$	80.0 ± 25.7	119 ± 20	0.0002
Number of lamellae	64.4 ± 16.4	62.0 ± 15.1	0.4006
Interlamellar distance	16.9 ± 3.6	16.5 ± 5.7	0.1749
Elastic lamella thickness	5.03 ± 1.40	4.67 ± 0.87	0.7239
Elastin medial fraction (%)	25.5 ± 5.4	21.3 ± 10.4	0.1909
Cytoplasm medial fraction (%)	29.9 ± 6.3	31.8 ± 5.5	0.4357
Collagen medial fraction (%)	8.26 ± 5.88	8.15 ± 9.70	0.3375
GAG medial fraction (%)	7.12 ± 3.80	8.35 ± 3.68	0.2449
Elastin per x-sec (mm^2)	41.0 ± 6.5	44.4 ± 23.1	0.8322
Cytoplasm per x-sec (mm^2)	49.7 ± 14.5	65.7 ± 14.3	0.0135
Collagen per x-sec (mm^2)	14.0 ± 11.4	16.4 ± 17.3	0.6888
GAG per x-sec (mm^2)	11.8 ± 7.7	17.2 ± 7.5	0.0642

*Ascending aorta specimens from organ donors (nondilated) and patients undergoing aneurysm repair (aneurysm) were analyzed by histomorphometry. Subjects ≤ 40 years old were excluded for the subgroup analysis. Continuous variables are represented as mean ± SD and comparisons between groups are by Mann-Whitney test. GAG: glycosaminoglycans, x-sec: cross-section.

Supplementary Table S4: Influence of age on histomorphometry parameters in nondilated aortas*

	Nondilated ≤ 40 yr<br>*n* = 7	Nondilated > 40 yr<br>*n* = 10	*P* value
Age	29.6 ± 8.7	62.0 ± 8.2	<0.0001
Aorta diameter (cm)	2.96 ± 0.46	3.34 ± 0.29	0.0904
Aorta z-score	-0.44 ± 1.31	-0.13 ± 0.86	0.3638
Intima thickness (mm)	0.06 ± 0.05	0.22 ± 0.44	0.1331
Media thickness (mm)	1.53 ± 0.23	1.67 ± 0.25	0.3137
Adventitia thickness (mm)	0.15 ± 0.11	0.15 ± 0.14	>0.99
Media x-sec area (mm^2)	134 ± 32	164 ± 27	0.0431
SMC per mm^2	659 ± 96	481 ± 126	0.0043
SMC per x-sec x10^3	86.0 ± 11.4	80.0 ± 25.7	0.3638
Number of lamellae	72.3 ± 18.2	64.4 ± 16.4	0.5206
Interlamellar distance	13.8 ± 1.6	16.9 ± 3.6	0.0250
Elastic lamella thickness	4.74 ± 0.62	5.03 ± 1.40	0.9623
Elastin medial fraction (%)	38.1 ± 12.0	25.5 ± 5.4	0.0068
Cytoplasm medial fraction (%)	23.2 ± 8.2	29.9 ± 6.3	0.1331
Collagen medial fraction (%)	6.51 ± 7.80	8.26 ± 5.88	0.1613
GAG medial fraction (%)	7.65 ± 6.10	7.12 ± 3.80	>0.99
Elastin per x-sec (mm^2)	49.9 ± 15.2	41.0 ± 6.5	0.4173
Cytoplasm per x-sec (mm^2)	31.0 ± 12.7	49.7 ± 14.5	0.0250
Collagen per x-sec (mm^2)	8.07 ± 9.92	14.05 ± 11.40	0.0878
GAG per x-sec (mm^2)	10.9 ± 9.0	11.8 ± 7.7	0.6009

*Nondilated ascending aortas of young (≤ 40 years old) and older (> 40 years old) organ donors were analyzed by histomorphometry. Continuous variables are represented as mean ± SD and comparisons between groups are by Mann-Whitney test. GAG: glycosaminoglycans, x-sec: cross-section.

Supplementary Table S5: Influence of aortic valve morphology on histomorphometry parameters in aneurysmal aortas*.

	Aneurysm BAV<br>*n* = 9	Aneurysm TAV<br>*n* = 9	*P* value
Age	58.8 ± 8.9	66.6 ± 11.9	0.1538
Aorta diameter (cm)	5.33 ± 0.49	5.06 ± 0.41	0.2262
Aorta z-score	5.76 ± 1.84	5.36 ± 1.79	0.8633
Intima thickness (mm)	0.09 ± 0.12	0.33 ± 0.48	0.0400
Media thickness (mm)	1.28 ± 0.22	1.33 ± 0.16	0.5303
Adventitia thickness (mm)	0.07 ± 0.02	0.12 ± 0.08	0.1615
Medial x-sec area (mm^2)	209 ± 38	204 ± 21	0.9314
SMC per mm^2	551 ± 54	603 ± 67	0.0745
SMC per x-sec x10^3	114 ± 218	123 ± 21	0.4363
Number of lamellae	65.3 ± 10.7	58.7 ± 18.5	0.5280
Interlamellar distance	15.2 ± 2.0	17.7 ± 7.9	0.7304
Elastic lamella thickness	4.69 ± 0.87	4.65 ± 0.92	0.9314
Elastin medial fraction (%)	26.2 ± 8.2	16.4 ± 10.5	0.0244
Cytoplasm medial fraction (%)	29.9 ± 5.2	33.7 ± 5.5	0.1615
Collagen medial fraction (%)	6.80 ± 5.75	9.51 ± 12.76	0.4894
GAG medial fraction (%)	7.78 ± 3.72	8.92 ± 3.77	0.5457
Elastin per x-sec (mm^2)	54.5 ± 19.7	34.2 ± 22.6	0.0400
Cytoplasm per x-sec (mm^2)	63.0 ± 17.8	68.4 ± 10.0	0.2973
Collagen per x-sec (mm^2)	14.9 ± 12.9	18.0 ± 21.6	0.5457
GAG per x-sec (mm^2)	15.8 ± 6.9	18.6 ± 8.2	0.3401

*Ascending aorta specimens from patients with bicuspid (BAV) or tricuspid aortic valves (TAV) undergoing aneurysm repair were analyzed by histomorphometry. Continuous variables are represented as mean ± SD and comparisons between groups are by Mann-Whitney test. GAG: glycosaminoglycans, x-sec: cross-section.
